# Fabrication of porous silicon nanowires by MACE method in HF/H_2_O_2_/AgNO_3_ system at room temperature

**DOI:** 10.1186/1556-276X-9-196

**Published:** 2014-04-30

**Authors:** Shaoyuan Li, Wenhui Ma, Yang Zhou, Xiuhua Chen, Yongyin Xiao, Mingyu Ma, Wenjie Zhu, Feng Wei

**Affiliations:** 1 State Key Laboratory of Complex Nonferrous Metal Resources Clear Utilization/Silicon Metallurgy and Silicon Material Engineering Research Center of Universities in Yunnan Province, Kunming 650093, China; 2 Faculty of Metallurgical and Energy Engineering, Kunming University of Science and Technology, Kunming 650093, China; 3 Faculty of Physical Science and Technology, Yunnan University, Kunming 650091, China

**Keywords:** Porous silicon nanowires, Lightly doped Si substrate, ‘One-pot procedure’ MACE, Self-electrophoresis model

## Abstract

In this paper, the moderately and lightly doped porous silicon nanowires (PSiNWs) were fabricated by the ‘one-pot procedure’ metal-assisted chemical etching (MACE) method in the HF/H_2_O_2_/AgNO_3_ system at room temperature. The effects of H_2_O_2_ concentration on the nanostructure of silicon nanowires (SiNWs) were investigated. The experimental results indicate that porous structure can be introduced by the addition of H_2_O_2_ and the pore structure could be controlled by adjusting the concentration of H_2_O_2_. The H_2_O_2_ species replaces Ag^+^ as the oxidant and the Ag nanoparticles work as catalyst during the etching. And the concentration of H_2_O_2_ influences the nucleation and motility of Ag particles, which leads to formation of different porous structure within the nanowires. A mechanism based on the lateral etching which is catalyzed by Ag particles under the motivation by H_2_O_2_ reduction is proposed to explain the PSiNWs formation.

## Background

Silicon nanowires (SiNWs) have widely attracted attention due to their unique physical and chemical properties and potential applications in optoelectronics [1], thermoelectrics [2,3], energy conversion and storage [4-6], and biomedicine [7,8]. Numerous methods have been developed to fabricate SiNWs including bottom-up or top-down technologies, such as vapor-liquid–solid growth [9,10], solid–liquid–solid growth [11,12], reactive ion etching [13], or metal-assisted chemical etching (MACE) [14]. Compared with the other techniques, the MACE is a simple and low-cost method offering better structure controllability of silicon nanowire such as diameter, length, orientation, morphology and porosity, which, therefore, has attracted increasingly research interests in the past decade [5,14,15]. In principle, the MACE process includes two successive steps, the nucleation of metal catalysts and anisotropic etching, which are classified as the one-step and two-step MACE, respectively [16]. In the one-step MACE (1-MACE), the two processes take place in an etching solution containing HF and metal salts. In the two-step MACE (2-MACE), metal catalysts are firstly deposited on the wafer surface, and the subsequent anisotropic etching occurs in the HF/oxidant (oxidant = H_2_O_2_[17,18], Fe(NO_3_)_3_[19,20] or KMnO_4_[21], etc.) solution.

Recently, the fabrications of one-dimensional silicon nanowires with porous structure using the MACE method have been given more wide attention. The emerging mesoporous silicon nanowires (MPSiNWs) open a new door to develop the wide applications derived from the enhanced surface areas and quantum confinement effect [22]. The doped type and concentration, fabrication methods and etching temperature have an important effect on the morphology of silicon nanowire. Yang et al. [23] have reported that the MPSiNWs were fabricated by 1-MACE with highly doped p-type silicon at temperature of 25°C to 50°C. To et al. [22] reported that the MPSiNWs were also obtained by etching highly doped n-type silicon with the 1-MACE method. In addition, the 2-MACE was also often reported to fabricate PSiNWs [24-27]. In general, it has been found that the roughness of silicon nanowires is increased with increasing doped level and H_2_O_2_ concentration [24,28]. For both MACE, the lightly doped silicon wafers are often difficult to obtain PSiNWs [22-27].

In the present work, the H_2_O_2_ oxidant was introduced into HF/AgNO_3_ etching solution for fabricating PSiNWs, which might be called ‘one-pot procedure’ MACE, it is practicable method for fabricating PSiNWs, even for lightly doped ones. The effect of doped level on nanostructure of SiNWs was studied. Meanwhile, the effects of H_2_O_2_ concentration on nanostructure of lightly doped SiNWs were also investigated. According to the experiment results, a model was proposed to describe the pore formation process.

## Methods

The moderately and lightly doped p-type Si(100) wafers with resistivity of 0.01 ~ 0.09 and 10 ~ 20 Ωcm were respectively selected as the starting wafer. Prior to etching, the wafers were cut into 1 × 1 cm^2^, and then were cleaned by ultrasonication in acetone, ethanol, and deionized water, respectively. The clean silicon wafers were immersed into dilute HF solution to remove the native oxide layers and result in a hydrogen-terminated surface. The etching process was carried out by fixing the cleaned wafers in a plastic beaker which held the etchant solution containing 4.6 mol/L HF, 0.02 mol/L AgNO_3_, and H_2_O_2_ with different concentrations (0, 0.03, 0.1, 0.4, 0.8 mol/L). The etching was operated for 60 min under ambient temperature in the dark room. After etching, the samples were immediately dipped into 50 wt.% HNO_3_ to dissolve the as-generated Ag dendrites. Finally, the wafers were thoroughly rinsed with deionized water and dried by N_2_ blowing.

The physical morphology of SiNWs was characterized by scanning electron microscopy (SEM; QUANTA200, FEI, Hillsboro, OR, USA) and transmission electron microscopy (TEM; JEM-2100, JEOL, Akishima-shi, Japan). The crystallinity was studied by selected-area electron diffraction (SAED, integrated with JEM-2100 TEM). For the TEM, high-resolution TEM (HRTEM), and SAED analyses, SiNWs were scratched off from the substrates and spread into ethanol and then salvaged with copper grids. The characterizations were performed under the voltage of 200 kV.

## Results and discussion

**Figure 1:**
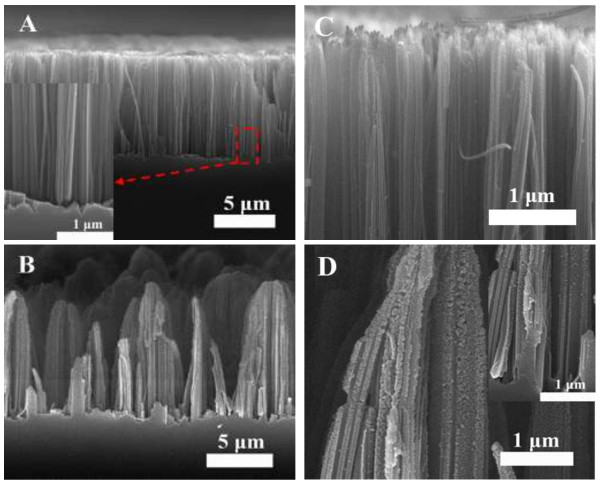
**Cross-sectional images of SiNWs from moderately doped silicon wafer under various concentration of H**_**2**_**O**_**2**_**. (A,C)** 0 and **(B,D)** 0.03 mol/L. The insets in **A** and **D** show the roots images of SiNWs.

Figure 1 displays the cross-sectional SEM images of as-prepared medially doped SiNWs. The large-scale image of Figure 1A shows that the SiNWs from HF/AgNO_3_ system are dense and in an orderly and vertical orientation. The uniform lengths of these SiNWs are about 10 μm and their diameters are about 100 ~ 200 nm. The roots of SiNWs show solid and smooth surface, as shown in the inset. But the top of the SiNWs shows a slightly porous structure. The pores are induced by Ag^+^ ion nucleation and dissolution of Si, which has been reported by previous researcher [24]. The Ag^+^ ion concentration is increased from root to top of SiNWs, leading to an increasing nucleation and Si oxidization, which can be used to explain why the top of nanowire is porous [28]. However, SiNWs show an obvious morphology difference when H_2_O_2_ is introduced into the HF/AgNO_3_ system, the top of the nanowires gather together, which could be attributed to the degenerate rigidity and increased strain with the presence of numerous porous structures [23,29]. From the corresponding magnified images in Figure 1D, we can find that the whole of the nanowire is covered by numerous porous structures. Numerous generated Ag^+^ ions could spread throughout the SiNWs, and subsequently nucleate on the surface of SiNWs, under the catalysis of Ag nanoparticles, the pore structures would be formed around the nanowire. Meanwhile, the density of SiNWs is decreased by comparing with that of Figure 1A, it agrees with the results reported by Zhang et al. [25], and which is attributed to excessive dissolution of Si. The lengths of SiNWs are not very uniform, but most of them have lengths of about 11 μm and are longer than that of Figure 1A. It indicates that the reaction driving force is larger in this case.

**Figure 2:**
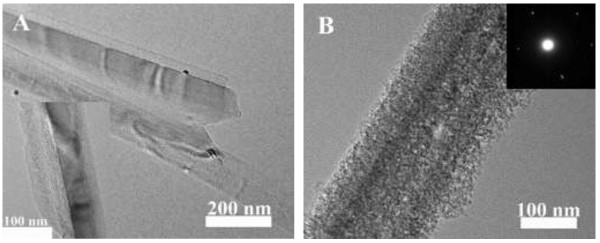
**TEM images of SiNWs from moderately doped silicon wafer under various concentration of H**_**2**_**O**_**2**_**. (A)** is the root of SiNWs prepared under etchant with 0 mol/L H_2_O_2_; the inset is the top of SiNWs. **(B)** is prepared under etchant with 0.03 mol/L H_2_O_2_; the inset shows the SAED pattern.

The TEM characterizations were used to further study nanostructure and crystallinity of PSiNWs. The typical TEM images were shown in Figure 2. The SiNWs show solid roots and rough top, which is respectively shown in Figure 2A and in the inset. When the etchant contains H_2_O_2_, the SiNWs surfaces are covered by numerous mesoporous structure with diameters of about 5 ~ 10 nm. The SAED pattern shows that the MPSiNWs still keep a single crystalline structure.

The lightly doped wafer was also selected as the starting material besides medially doped silicon substrate. The H_2_O_2_ plays an important role in fabricating SiNWs through the 2-MACE process, which affects not only the etching rate, but also the morphology, nanostructure, and orientation of SiNWs [24,25,30,31]. Thus, in the HF/AgNO_3_/H_2_O_2_ system, the effect of H_2_O_2_ concentration on the nanostructure of lightly doped SiNWs was carefully studied in this part.

After the etching, some silver dendrites formed and covered the wafer, and their sizes were decreased with the increasing H_2_O_2_ concentration. Meanwhile, the color of Ag dendrite changed regularly with the increase of H_2_O_2_. Without H_2_O_2_, the Ag dendrite showed a grey and black, which might be caused by the formation of silver oxide. The dendrite color became shinning silver-white with the increase of H_2_O_2_. The above results indicate that the Ag dendrite can be oxidized into Ag^+^ by H_2_O_2_ according to the following reaction:

(1) $$2\mathrm{Ag}+H_{2}O_{2}+2H^{+}\to 2{\mathrm{Ag}}^{+}+2H_{2}O\;E^{0}=1.77V\;\left(\mathrm{vs}.\mathrm{SHE}\right)$$

**Figure 3:**
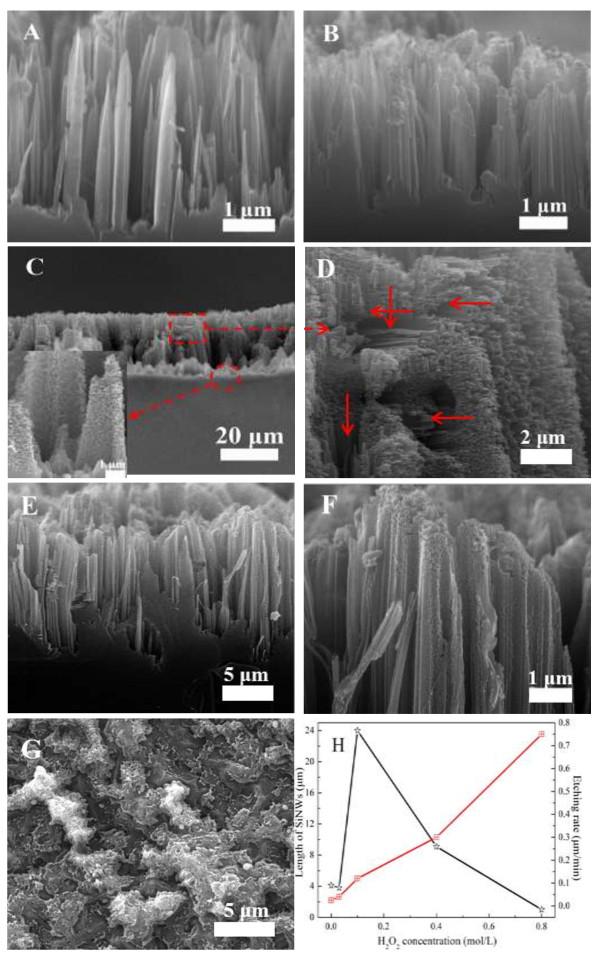
**SEM images of etched lightly doped silicon wafer under various concentration of H**_**2**_**O**_**2**_**. (A)** 0, **(B)** 0.03, **(C,D)** 0.1, **(E,F)** 0.4, and **(G)** 0.8 mol/L. **(H)** The lengths of SiNWs and etching rates as function of H_2_O_2_ concentration. The inset in **(C)** shows the magnified image of SiNWs, the part in the dotted box is magnified in **(D)** and the pore channels are marked as red arrows.

It can be found that the SiNW structure and morphology are severely affected by the doping levels of wafers by comparing the experiment results in Figures 1 and 3. When the etchant solution has no H_2_O_2_, the resulting lightly doped SiNW arrays show sharp top and smooth surface; the length (about 4 μm) is shorter and denser than that of the medially doped one, which indicates that the higher doping level is beneficial for SiNW growth and porosity formation, and also for SiNWs from the HF/H_2_O_2_/AgNO_3_ system (by comparing with Figures 1B and 3B). As we know, both Ag^+^/Ag or H_2_O_2_/H_2_O couples have higher positive equilibrium potentials than silicon E_VB_. Thus, the holes will be injected into the valence band of silicon with the Ag deposition or reduction of H_2_O_2_, which induces silicon substrate oxidization and dissolution, leading to SiNW growth and porosity formation.

**Figure 4:**
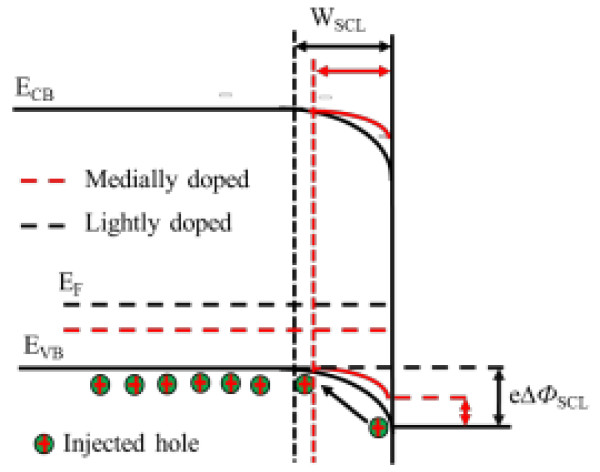
**The energy band diagram for p-type silicon in contact with etching solution.** The Schottky energy barrier (e∆*Ф*_SCL_) form with the build of energy equilibrium between silicon and etching solution.

Figure 4 shows the energy band diagram for p-type silicon in contact with etching solution. Under equilibrium conditions, the Fermi energy in silicon is aligned with the equilibrium energy of etching solution, resulting in the formation of a Schottky barrier that inhibits charge transfer (holes injection) across the interface [32]. The heavier dopant concentrations (i.e., lower Fermi level) will cause the bands to bend less and decrease the space charge layer width (W_SCL_) and the energy barrier (e∆Ф_SCL_) at the surface. Under the same etching conditions, a lower energy barrier will increase silicon oxidization and dissolution, thus accelerate SiNW growth or pore formation [23]. Furthermore, a higher dopant concentration of the silicon wafer would result in a higher crystal defects and impurities at the silicon surface which is considered as nucleation sites for pore formation [33].

With the presence of H_2_O_2_ in etchant, the etch rate is increased, and the nanowires become rough or porous; it may be attributed to the more positive redox potential of H_2_O_2_ (1.77 V vs. standard hydrogen electrode (SHE)) than that of Ag^+^ (0.78 V vs. SHE), which can more easily inject hole into the Si valence band through the Ag particle surface.

(2) $$H_{2}O_{2}+2H^{+}\to 2H_{2}O+2h^{+}$$

The H_2_O_2_ would be quickly exhausted by reactions 1 and 2 during the growth of nanowires, when the concentration is too low (e.g., 0.03 mol/L); thus, the change of etch thickness is not very remarkable. When the H_2_O_2_ concentration is 0.1 mol/L, the etching is significantly increased and the length of nanowire dramatically increases to about 24 μm. The Ag nanoparticles dramatically enhance the etching by catalyzing the sufficient H_2_O_2_ reduction [34]. Meanwhile, it can be found that the whole SiNWs are covered by numerous macroporous structures (as shown in the inset), which brings a poor rigidity and leads some damage during the cutting process. From the magnified images in Figure 3B, numerous lateral etched pore channels can be found, which indicates that some large-sized Ag particles nucleate throughout nanowires and laterally etch the nanowire. The length of SiNWs is sharply decreased with the increase of H_2_O_2_ concentration, and the PSiNWs show flat-topped structure, which may be attributed to the top oxidation and dissolution of SiNWs. It indicates that the growth of SiNWs is the result of competition between lateral and longitudinal etching. When H_2_O_2_ concentration increases to 0.8 mol/L, the sample with gray-white etched surface can be obtained. Some etch pits can be observed on the surface, it may be attributed to the SiNWs polishing induced by excessive H_2_O_2_. Silicon chemical etching in HF solution containing oxidant species is known to be a mixed electroless and chemical process [35]. The polishing mechanism of Si in the low-ratio HF/H_2_O_2_ system can be described by the following reaction [34]:

(3) $$\mathrm{Si}+6\mathrm{HF}+2H_{2}O_{2}\to H_{2}{\mathrm{SiF}}_{6}+2H_{2}O$$

The SiNW length and etching rate evolution vs. H_2_O_2_ concentration were summarized, the etching rates were calculated according to the formula *R* = ∆*m*/*d*_Si_*St*[34]. The quantity of dissolved silicon (mass loss, ∆*m*) is obtained by weighting the silicon wafer before and after the etching, the density of silicon (*d*_Si_) is 2.33 g/cm^3^, the area of the wafer (*S*) is 1 × 1 cm^2^, and etching time (*t*) is 60 min; the results were shown in Figure 3H. A nonmonotonic trend in SiNW length evolution with increasing H_2_O_2_ concentration is observed, and which belies the monotonic increasing etching rate. It is caused by the increasing top lateral etching with increasing H_2_O_2_ concentration.

**Figure 5:**
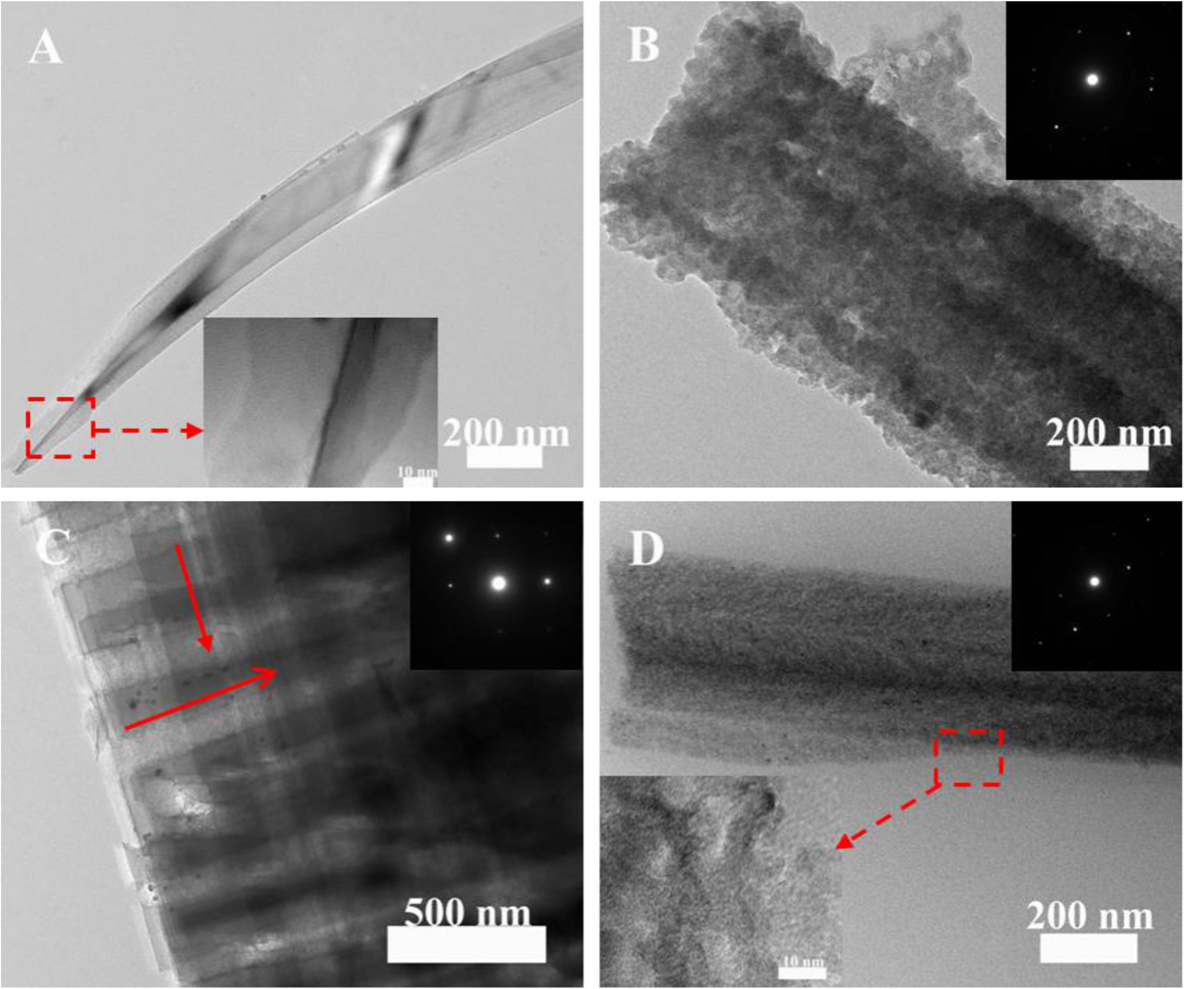
**TEM images (A,B,C,D) of lightly doped silicon nanowires under various concentration of H**_**2**_**O**_**2**_**. (A)** 0,** (B)** 0.03, **(C)** 0.1, **(D)** 0.4 mol/L.

According to the above TEM results, we can find that the nanostructures of SiNWs have been affected by the concentration of H_2_O_2_. It can be seen that the lightly doped SiNWs from the HF/AgNO_3_ system show a tapering top and solid surface, as shown in the inset. With the addition of H_2_O_2_, the rough and porous silicon nanowires can be obtained, When H_2_O_2_ concentration is 0.1 mol/L, numerous almost perpendicular pore channels, with diameter about 100 nm, can be observed in the etched silicon (as shown in Figure 5C), which may be caused by the strong lateral etching driven by the reduction of H_2_O_2_. It can be found that mesoporous structures arise again when the H_2_O_2_ concentration increases to 0.4 mol/L. It indicates that H_2_O_2_ concentration plays a key impact on the size of renucleated silver particle and etching behaviors of SiNWs, which finally leads different porous structure within the nanowires. The high H_2_O_2_ concentration would be favorable to form Ag particles with small sizes which are responsible for the formation of mesoporous structures within SiNWs [24]. From the HRTEM characterization in Figure 5D, some etching pits and pores, with the size of about 5 ~ 10 nm, can be observed on the surface of SiNWs. The SAED characterizations indicate all of the porous silicon still keep a single crystalline structure. The above results demonstrate that the size of Ag particles formed through renucleation is influenced by H_2_O_2_ species, which in turn affect the nanostructure of SiNWs.

The self-electrophoresis mode proposed by Peng et al. [18] describe the Ag particle migration under the drive by H_2_O_2_ reduction, which can be used to explain the perpendicular longitudinal and lateral etching phenomenon in the MACE process. It shows that the motility of Ag particles in Si is associated with catalytic conversion of chemical free energy into propulsive mechanical power. On the surface of silicon, the generated Ag nanoparticles work as microcathodes, which catalyze H_2_O_2_ reduction at the surface facing the etchant, consuming proton (H^+^) and electrons in the process (reaction 4). The other side of Ag particle facing the Si would works as the catalyst to oxidize Si and generate electron, which generate H^+^ and electrons (reaction 6). The reactions at cathode (Ag facing the electrolyte) and the anode (Si contacting with Ag) sites are outlined as follow [14].

(4) $$\begin{array}{l} \mathrm{Cathode}\;\mathrm{reaction}:\;H_{2}O_{2}+2H^{+}\to 2H_{2}O\\ \;+2h^{+}\;E^{0}=1.77\;V\;\left(\mathrm{vs}.\mathrm{SHE}\right) \end{array}$$

(5) $$2H^{+}+2e^{-}\to H_{2}↑$$

(6) $$\begin{array}{l} \mathrm{Anode}\;\mathrm{reaction}:\mathrm{Si}+4h^{+}+6\mathrm{HF}\to H_{2}{\mathrm{SiF}}_{6}\\ \;+4H^{+}\;E^{0}=1.2\;V\;\left(\mathrm{vs}.\mathrm{SHE}\right) \end{array}$$

(7) $$\mathrm{Overall}\;\mathrm{reaction}:\mathrm{Si}+H_{2}O_{2}+6\mathrm{HF}\to 2H_{2}O+H_{2}{\mathrm{SiF}}_{6}+H_{2}↑$$

**Figure 6:**
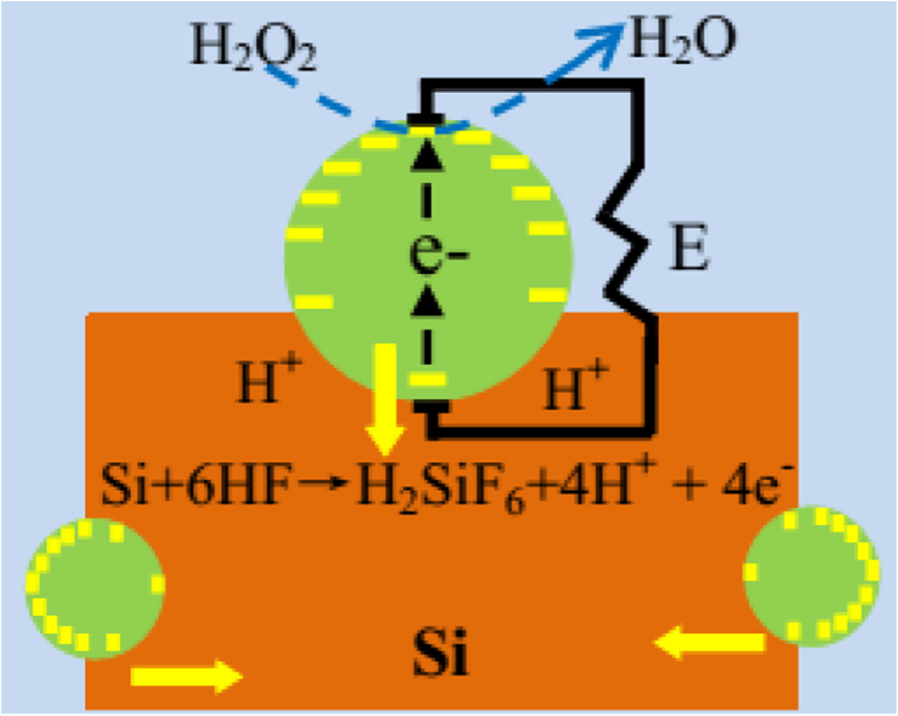
**Ag particle migration in bulk Si driven by self-electrophoresis mode.** An electric field is built with the presence of H^+^ gradient across the Ag particle from the anode site to cathode site, which can propel Ag particles toward the anode site.

The potential of the cathode site (EH_2_O_2_ = 1.77 V vs. SHE) is higher than that of the anode site (E_Si_ =1.2 V vs. SHE), thus a local corrosion current would flow from the cathode site to the anode site. In this case, the catalytic Ag particle would work as a redox center and act as a short-circuited galvanic cell with an electron flow inside the Ag particle, while H^+^ would migrate outside the Ag particle from the anode site to the cathode site. The H^+^ gradient across the Ag particle from the anode site to cathode site would build-up of an electric field which would propel Ag particles (with negative charge) toward the anode site, thus, the Ag particles deposited on the surface and side of SiNWs would migrate in a vertical or horizontal direction, respectively, as shown by the yellow arrows in Figure 6. It can satisfactorily explain the perpendicular longitudinal and lateral etching pore channel in Figure 5C.

**Figure 7:**
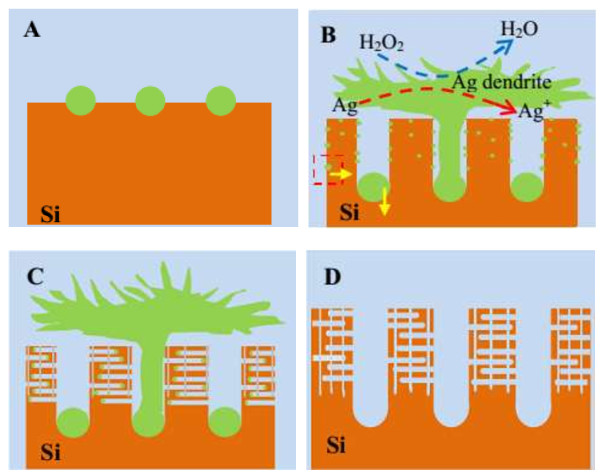
**Schematic illustration of the formation process of PSiNWs through MACE method in HF/H**_**2**_**O**_**2**_**/AgNO**_**3 **_**system. (A)** Ag nanoparticles deposit on silicon surface at the beginning. **(B)** SiNWs grow with the migration of Ag particle, and some Ag^+^ ions renucleate throughout the nanowires. **(C)** Numerous perpendicular pore channel form with the migration of renucleated Ag particle. **(D)** Porous structure can be obtained with the removal of Ag^0^.

The formation process of mesoporous structures within the SiNWs may be consistent with that of macroporous structures, both are caused by the lateral etching of silicon, i.e., lateral motility of Ag particles. The four steps are proposed to describe the PSiNWs formation in the HF/AgNO_3_/H_2_O_2_ etching system. When silicon wafers were immersed into the etchant, Ag nanoparticles were deposited on silicon surface, as depicted in Figure 7A. According to the self-electrophoresis mode, the nucleated Ag particles would migrate down and form the SiNWs, the duration of the redox reaction couple of reactions 4 and 6 lead to the growth of SiNWs. In addition, the reaction of silver ion deposition (Ag^+^ + e^−^ → Ag) is still present during the growth of SiNWs. Thus, some of the silver particles would grow into dendrite and cover the surface of SiNWs, just as Ag dendrite form in the one-step MACE [28]. As the standard reduction potential of H_2_O_2_ (1.77 eV) is larger than that of Ag (0.78 eV), the growing Ag dendritic layer can simultaneously be oxidized into Ag^+^ ions by H_2_O_2_ (reaction 2). The generated Ag^+^ ions could renucleate throughout the nanowires, as shown in Figure 7B. The horizontal and vertical migrations of Ag particles driven by self-electrophoresis finally induce perpendicular pore channels (Figure 7C). The porous structure can be obtained after the Ag^0^ removal by concentrated nitric acid (Figure 7D). The concentration of H_2_O_2_ influences the nucleation and motility of Ag particles, which leads to the formation of different porous structures within the nanowires. When H_2_O_2_ concentration is too high, the excessive Ag^+^ would be produced, and they renucleate to form numerous Ag particles which catalyze H_2_O_2_ reduction and induce excessive silicon dissolution. That is to say, the polishing would be induced under high H_2_O_2_ concentration of the HF/AgNO_3_/H_2_O_2_ system.

## Conclusion

This work has demonstrated a simple MACE method for successfully fabricating lightly doped porous silicon nanowires at room temperature. The effects of H_2_O_2_ concentration on nanostructure of moderately and lightly doped SiNWs were investigated. The results indicate that the concentration of H_2_O_2_ influences the nucleation and motility of Ag particles, which leads different porous structure within the nanowires. In the HF/AgNO_3_/H_2_O_2_ etching system, the H_2_O_2_ species replaces Ag^+^ as the oxidant and the Ag nanoparticles work as catalyst during the etching. A mechanism based on the lateral etching which is catalyzed by Ag particles with the motivation of H_2_O_2_ reduction is proposed to explain the formation of PSiNWs. The simple etching system not only synthesizes large-scale moderately doped single crystalline PSiNWs, but can also fabricate lightly doped ones, which can open up exciting opportunities in a wide range of applications. For example, the vertically aligned nanowires with a high surface area can be exploited as a high-capacity electrode for supercapacitors. The deep quantum confinement effect and biodegradability feature of the porous silicon nanowires may enable interesting applications in optoelectronics and drug delivery.

## Competing interests

The authors declare that they have no competing interests.

## Authors’ contributions

SL designed the experiment, analyzed results, and drafted the manuscript. WM and YZ offered financial support. XC and YX offered technical supports. MM, WZ, and FW participated in revising the manuscript. All authors read and approved the final manuscript.
